# Evaluation of a Technological Image-Based Dietary Assessment Tool for Children during Pubertal Growth: A Pilot Study

**DOI:** 10.3390/nu11102527

**Published:** 2019-10-20

**Authors:** Jiao-Syuan Wang, Rong-Hong Hsieh, Yu-Tang Tung, Yue-Hwa Chen, Chen Yang, Yang Ching Chen

**Affiliations:** 1 Department of Family Medicine, Taipei Medical University Hospital, Taipei 110, Taiwan; mm07110703@gmail.com; 2 School of Nutrition and Health Sciences, College of Nutrition, Taipei Medical University, Taipei 110, Taiwan; hsiehrh@tmu.edu.tw (R.-H.H.); yuehwa@tmu.edu.tw (Y.-H.C.); 3 Graduate Institute of Metabolism and Obesity Sciences, College of Nutrition, Taipei Medical University, Taipei 110, Taiwan; f91625059@tmu.edu.tw; 4 School of Food Safety, College of Nutrition, Taipei Medical University, Taipei 110, Taiwan; 5 Department of Pediatrics, Taipei Medical University Hospital, Taipei Medical University, Taipei 110, Taiwan; yeungsann@yahoo.com.tw; 6 Department of Family Medicine, School of Medicine, College of Medicine, Taipei Medical University, Taipei 110, Taiwan

**Keywords:** image-based dietary assessment, puberty, food records, macronutrients, micronutrients

## Abstract

We designed an image-based dietary assessment tool called COFIT, which means “fit together” and pilot-tested it in the Taipei Puberty Longitudinal Study (TPLS). Children aged 6–17 years were invited to use COFIT over three days for recording all instances of eating in addition to maintaining written food records (FR). Spearman’s correlation and Bland–Altman analysis were used to compare the intake of macronutrients and micronutrients estimated using the image-based dietary assessment and the FR method. Intra-class correlation coefficients were used to estimate reliability between dietitians. In the final analysis, 23 children (mean age: 10.47 ± 0.47 years) with complete data obtained using two dietary assessment methods were included. Reliability among dietitians was high. Most assessments of macronutrients and micronutrients revealed moderate correlations between the two methods (range: 0.27–0.94); moreover, no significant differences in nutrients assessments were observed between the two methods, except for energy and fat. The average difference in energy intake between the methods was 194 kcal/day. Most limits of agreement were within an acceptable range. The Bland–Altman plots showed robust agreement with minimum bias. The limitation was the small sample size and not dividing the population into children and teenagers since the two groups may have different food consumption habits. Overall, the results showed that the image-based assessment tool is suitable for assessing children’s dietary intake of macronutrients and micronutrients during pubertal growth.

## 1. Introduction

Adequate and balanced nutrition is essential for the pubertal growth of children and adolescents [1,2]. However, the precise assessment of the nutritional intake of young children remains a challenge in clinical practice. Previous studies have indicated that traditional methods, such as 24-h dietary recall (24-HDR) and food records (FR), may be limited by recall bias [3,4,5]. Children may not recognize ingredients and may not accurately record what they have eaten. From the self-reported dietary recall of the children themselves, the dietitian may not be able to accurately record “real-time” portion sizes, which increases the risk of misreporting nutrient intake [6]. Therefore, 24-HDR is frequently completed by children’s primary caregivers, but they often fail to capture the actual 24-h dietary intake of their children, particularly their intake at school during lunch [4,7]. If children need to spend a long time memorizing and documenting food, it may increase respondent burden [5]. Food-frequency questionnaires have also been reported to cause higher estimation errors than other traditional self-reporting methods [8,9]. Therefore, long-term nutrient assessment is difficult. Thus, a simple and convenient approach to monitor nutrient intake is necessary for children.

In recent years, various image-based methods for creating FR have been developed [10], and image-based dietary assessment applications are becoming increasingly popular. Technology-based FR enables users to record their food intake immediately, and they have high portability compared with traditional methods. Even illiterate individuals, such as preschool children, can easily use technology-based FR after proper training. Technology-based FR can be linked to a large-scale integrated database for semiautomatic identification of the source and brand of food, thus assisting dietitians to collect accurate dietary information [11]. By recording photographs of a meal before and after consumption, researchers can calculate the food and beverages consumed and estimate portion sizes directly from photographs. After that, researchers can send reports and feedback via the Internet to the participants. This approach can reduce costs as well as the workload of both participants and researchers [12,13].

Previous studies have suggested that image-based dietary assessment technology may have different degrees of acceptance among various study populations [5,14]. Ensuring the applicability of image-based dietary assessment tools to different groups is necessary [15]. Most previous studies have proved that image-based FR are useful for adults [16,17,18] and older people [19]. Research on the use of image-based nutritional assessment applications by children is relatively limited. One image-based application named the Tool for Energy Balance in Children (TECH) is designed for preschool children; however, its reliability and validity remain unclear because it is not accurate at the individual level but may become a potentially useful tool at the group level [20,21,22]. The energy intake measured by TECH was not significantly different from the ones assessed from the traditional dietary records. However, TECH showed poor accuracy to assess energy intake at the individual level from the wide limits of agreement in the Bland and Altman plot. Another study indicated that children might prefer to use electronic methods for nutrient data collection instead of written methods (75% vs. 50% compliance) [23]. Overall, the number of image-based dietary assessment applications designed for Asian populations is relatively low. Diverse dietary patterns in Asian cultures and complex cooking styles in Asian countries, such as Taiwan, pose challenges to written methods of accurately assessing nutrient intake. Traditional Chinese cooking style includes stir-frying, steaming, braising, Hong-shao, and roasting, which makes detailed nutrients assessment even more difficult. Thus, we developed a new image-based assessment application called COFIT (The name combined with the prefix “co-” and the root “fit”, hoping users could “become fit together”) for making FR, which can be used on a mobile phone. In order to solve previous problems in image-based application and capture the uniqueness in Asian cooking styles, COFIT incorporates text entry along with digital images to identify food. COFIT also provides a web-based interaction with dietitians and an integrated database for estimating energy or nutrients. These techniques assist dietitians in collecting dietary information more accurately. In this study, we aimed to launch a pilot study and established the preliminary validity and reliability of COFIT, targeting children at the pubertal growth stage. Furthermore, we compared the macronutrient and micronutrient assessments made using technology-based FR and those made using traditional written FR. Thus, we validated COFIT and determined whether COFIT can assist children and their primary caregivers in monitoring whether the children have balanced and sufficient nutrition intake.

## 2. Materials and Methods

Since July 2018, we have been conducting the Taipei Puberty Longitudinal Study (TPLS) by recruiting participants from the Department of Pediatrics at Taipei Medical University Hospital. Children who may have growth problems, such as developmental retardation, being overweight, or precocious puberty, tend to visit pediatricians for advice. We recruited children who were undergoing pubertal growth; the boys and girls were aged 9–17 and 6–14 years, respectively. We enrolled in our study, the children who had not received a diagnosis of developmental retardation. The study ethical protocol was approved by the Institutional Review Board of Taipei Medical University (N201802018) and complied with the principles outlined in the Helsinki Declaration.

When the participants were first enrolled in the study, we provided them an introduction to the study to ensure that they understood the process and provided informed consent. Next, we conducted a 20-min session to demonstrate how to write FR for three out of seven days. We requested that the FR detail the time and location of eating, the food and its ingredients, cooking method, seasoning, portion size, and other information that would assist the dietitians’ analysis, such as the brand of food or leftovers after a meal. These FR would be returned after one week when the participants came for a follow-up consultation. Dietitians would examine the content. If any data were missing, the participants were requested to provide it.

In addition, we assisted the participants in installing the proposed image-based dietary assessment application on their mobile phone and taught them to use the application on-site for 20 to 30 min. The participants were instructed to record seven days of FR and three days of COFIT within these seven-days. The three matching days (containing one weekday and two weekend days) of dietary records were used for analysis. We instructed the children to choose one weekday near the two chosen weekend days. Photos were expected to include three meals as well as snacks to help dietitians and physicians assess whether they received adequate nutrients in daily life. The basic requirements for recording photographs were as follows: (1) Before recording photographs, participants needed to keep other objects, such as spoons or coins, near their meals or snacks to provide a scale that could assist the dietitians to assess portion sizes. (2) The image was to be photographed at an angle of 45° to enable the dietitians to assess the height of the food [14,24,25]. (3) Food items containing stuffing, such as rice balls, buns, or spring rolls, were to be photographed in their prepared form and opened and photographed again to ensure ingredients of the stuffing were completely visible in the picture. (4) The commensal eating pattern is typical in Asia; however, assessing the actual portion size of an individual in this pattern is difficult. In order to assess the children’s actual food consumption, the parents were asked to appropriately plate food for the children before meals on the three days on which photographs were to be recorded. Only the photographs that met the aforementioned criteria were included in the statistical analysis. We ensured that both the children and parents knew how to use the application and record photographs correctly. The parents were allowed to assist young children if necessary. We then compared these two records to analyze relative reliability.

COFIT is an image-based dietary assessment application designed for both commercial and academic purposes that were initially developed jointly by computer scientists, professors of nutrition, and a group of highly experienced dietitians [15]. Two distinguishing features of COFIT are that it combines multiple functions and enables online consultation with dietitians. It allows users to key-in physical data, such as height, weight, percentage of body fat, and blood pressure, to the online integrated physiological database. By setting goals and reviewing progress regularly, participants can self-monitor their health and change over a long period. Moreover, the participants used photos to record their meals and additionally typed food descriptions in the text to provide additional information. Trained dietitians interpreted the participants’ image-based FR and sent nutrient analysis reports from the back-end server; the participants could also contact the dietitians through the system to ask them any detailed queries. This application requires an operating system, such as Android or IOS, and can be used free of cost.

After FR collection, two trained dietitians independently entered data from handwritten diaries and photographs into the system and disaggregated the foods into their constituent ingredients. Energy and nutrient intake were estimated on the basis of Nutritionist Edition, COFIT Pro, Version 1.0.0, a software package for nutrient analysis that features a Taiwanese food composition table as the nutrient database (Taipei, Taiwan) [26] and commercial food ingredients provided by food companies. Details of the disaggregated food items, such as portion size, total calorie count, and multiple macronutrients and micronutrients, were the output from the analysis. The intake of nutrients over three days was summed and averaged, and both data were recorded.

We classified nutrients like energy, macronutrients, and micronutrients for estimating intake. The energy was averaged from the collected three-days data and calculated as kcal/day. Macronutrients include protein, fat, carbohydrates, dietary fiber, and their basic units were calculated as grams. Moreover, crucial micronutrients, such as calcium, zinc, folate [1], phosphorus, iron, magnesium, potassium, and sodium, which affect children’s growth and development were also assessed, calculated as milligrams. The Wilcoxon signed-rank test was used to determine whether the differences between the two methods were statistically significant for non-parametric data. Spearman’s correlation coefficient analysis was used to investigate the correlations between these two methods, and coefficients >0.5 indicated a moderate degree of correlation [27]. To verify the between-rater variance in the nutritional assessments of the various dietitians, we selected three samples for intra-class correlation coefficient (ICC) analysis. The consistency between the two methods was assessed using Bland–Altman plots [28]. The average intakes determined using the methods were plotted on the *x*-axis, and the limits of the agreement, which were calculated as the mean difference ± 1.96 standard deviations, were plotted on the *y*-axis. SAS for Windows (version 9.4, 2014, SAS Institute, Cary, NC, USA) was used for analysis.

## 3. Results

A total of 33 children agreed to participate in this study. By December 2018, seven children had been lost to follow-up; consequently, we did not have their dietary intake data. Three participants did not have complete FR or adequate food photographs (two or fewer meals) and experienced difficulties in recalling the details of their meals; hence, we excluded them from the analysis. Finally, a total of 23 children with at least three days of complete written FR and image-based dietary assessment records were included in our analysis.

Table 1 shows the basic demographic characteristics of the 23 children; 56.5% (*n* = 13) of the children were boys and 43.5% (*n* = 10) were girls. The children’s body mass index (BMI) standards varied according to their age; hence, we classified BMI into four groups according to growth charts for Taiwanese children [29]. Three children were classified as overweight (13.04%) and two as obese (8.7%). Their mean age was 10.47 ± 3.36 years, and BMI was 18.41 ± 4.34 kg/m^2^.

Table 2 shows the macronutrients from FR, as assessed using the image-based assessment and FR. The average energy intake estimated by photographs was 194.23 kcal/day, which was significantly lower than that estimated using the FR method (1584.88 ± 369.03 kcal/day and 1779.11 ± 316.29 kcal/day, respectively. *p* ≤ 0.001). We also observed moderate correlation between the two methods (*r* = 0.64, 95% CI = 0.30–0.83, *p* < 0.01), and the ICCs in both methods were high (0.94 and 0.85). In the analysis of protein, we observed a significant but weak Spearman’s correlation coefficient (SCC; *r* = 0.44, *p* < 0.01) between the two methods and moderate ICCs (0.54, 0.68). In the analysis of fat, the SCC was good (*r* = 0.65, *p* < 0.01) between the two methods; however, the ICC was moderate in the image-based assessment method (0.65) but high in the FR (0.88). The contrary situation was observed for carbohydrates; a significant but weak SCC (*r* = 0.47, *p* = 0.03) was observed between the two methods, and a high and moderate ICC was observed in the image-based assessment method (0.98) and the FR (0.67), respectively. The analysis of sugar exhibited a good SCC between the two methods (*r* = 0.67, 95% CI = 0.35–0.85, *p* < 0.001) and also a high ICC between two dietitians (0.87, 0.89).

As evident in Table 3, we observed that Na had significant difference between the image-based assessment and FR method when Na was analyzed using the Wilcoxon signed-rank test. The ICCs for all micronutrients in the two methods were high, indicating no obvious intra-rater difference between the dietitians. The SCCs among the micronutrients, except for Mg and Zn, were significantly good. Na, Fe, and folic acid exhibited good SCCs (*r* = 0.66, *p* < 0.001; *r* = 0.52, *p* = 0.01; and *r* = 0.77, *p* < 0.001; respectively.), and Ca, K, and P exhibited significantly weak SCCs (*r* = 0.48, *p* = 0.02; *r* = 0.45, *p* = 0.03; and *r* = 0.43, *p* = 0.04; respectively).

**Figure 1:**
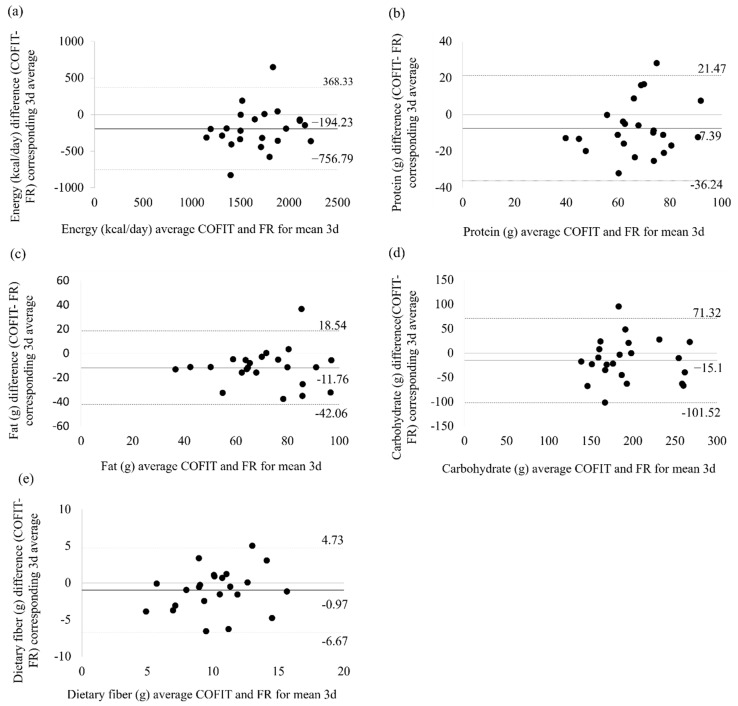
Macronutrients Bland–Altman plot for the mean of three days of COFIT and the mean of three days of FR. (**a**) Energy, (**b**) protein, (**c**) fat, (**d**) carbohydrates, and (**e**) dietary fiber.

**Figure 2:**
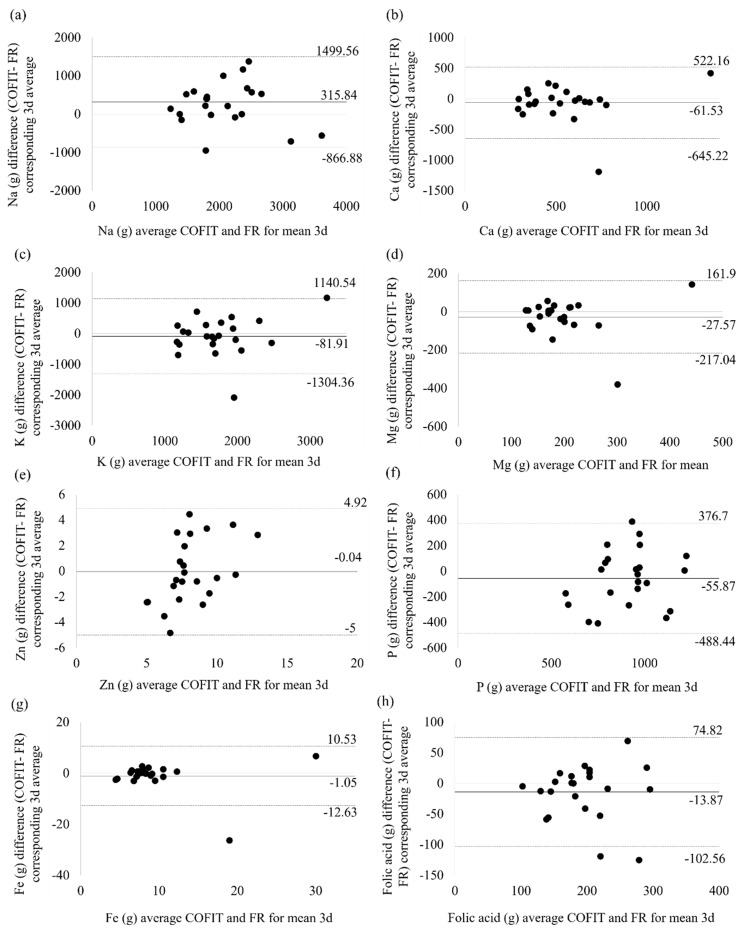
Micronutrients Bland–Altman plot for the mean of three days of COFIT and the mean of three days FR. (**a**) Na, (**b**) Ca, (**c**) K, (**d**) Mg, (**e**) Zn, (**f**) P, (**g**) Fe, and (**h**) folic acid.

Table 4 exhibits the mean difference in the intake of each nutrient as recorded using the image-based assessment and FR methods as well as the standard deviation and 95% limit of agreement (LOA) of the difference; the Bland–Altman plots are shown in Figure 1 and Figure 2. We observed an overestimate of 315.84 mg in Na in the image-based assessment compared with the FR method, with the 95% LOA ranging from −866.88 mg to 1499.56 mg. Most of the macronutrients and micronutrients were underestimated by the image-based assessment method. The magnitude of the mean difference in the macronutrient estimates ranged from −0.97 g for dietary fiber to −15.10 g for carbohydrate. The mean difference in the micronutrients ranged from −0.04 mg for Zn to −81.91 mg for K. We observed that most of the points approached the zero baselines (Figure 2), which indicated that these micronutrients have a high degree of consistency between image-based assessment and FR. Additionally, we observed outlier data for one child whose photographic report was consistently significantly underestimated compared with the FR (Figure 2). The highest mean difference, which was up to nearly 2000 mg between two methods, was found for K. We examined the original data and found that the high mean difference in K intake was observed because participants had reported very high vegetable intake in the FR method, but the portion size did not appear sufficiently large to substantiate the reported intake when the photographs were reviewed using COFIT.

## 4. Discussion

To our knowledge, COFIT is the first image-based dietary assessment application developed for research use in Asian countries. Our pilot study results proved the validity and reliability of using an image-based dietary assessment application for evaluating the essential macronutrients and multiple micronutrients that contribute to pubertal growth in children. In the image-based dietary assessment tool, which had food descriptions in the text, we successfully overcame the challenge posed by diverse dietary habits among Asian populations. Furthermore, we used multiple statistical methods to verify its validity, thus proving that it can be a reliable nutritional assessment tool for children during pubertal growth.

Correlations between the two assessment methods were moderate for energy, fat, sugar, Na, and folic acid and were acceptable for protein, carbohydrate, Ca, K, P, and Fe. Our results verified the feasibility of using image-based dietary assessment in nutritional research. We identified correlations in this validation study using the same scale; thus, we can compare our results to those of other similar studies. Our results were similar to those of one study [6], which showed strong associations in energy and fat estimates, but weaker correlations in protein estimates. In another study, which evaluated an application called NuDAM, the authors only observed weaker correlations for fat and sugar [25]. Our study reported adequate correlations between the two dietary assessment methods.

Similar to a previous study, we observed an underestimation of nutrition intake by the image-based dietary assessment compared with the traditional FR method. We believe that the major reason for the underestimation of calorie intake could be an underestimation of fat consumption. Fat constitutes a major part of total energy intake; however, it is not easily visible in pictures or detected by the eye. In our study, the image-based method underestimated intake by approximately 194 kcal/day in total energy, 7 g for protein, 15 g for carbohydrate, and 11 g for fat as compared with the intake assessed using the FR method. A review indicated that most image-based assessment tools have an underestimation tendency when compared with doubly labeled water (DLW) or another traditional method [15]. For example, Nutricam Dietary Assessment Method (NuDAM) [25], Remote Food Photography Method (RFPM) [30], and Technology Assisted Dietary Assessment (TADA) [17] differ in their underestimation of total energy intake, ranging from 179–895 kcal/day. Two other studies that used the TECH tool to assess children’s dietary intake reported that the underestimations were within 53–79 kcal of total energy expenditure (TEE) [21,22]. We observed similar results in our study with both photographs (1584 kcal/day, −18%) and FR (1779.11 kcal/day, −9%) yielding underestimated results compared with TEE (estimated 1950 kcal/day among children aged 10 years). We assumed such errors were due to both system and individual factors.

Other macronutrients and micronutrients were also underestimated compared with the dietary reference intake. We suspect that such differences resulted from system error or the following possible causes: (1) Participants may misreport their dietary intake, either by not recording all the food consumed or reducing their intake during the study period because they knew that they were being observed by researchers and dietitians [18,31]. (2) Foods with multiple ingredients or cooking methods may become challenging for food photography assessment. In our study, we found that specific types of foods could be the source of underestimation. For example, hot pot and sugary beverages, which have solid or soluble substances included in the base, as well as foods that were not optimally arranged on a plate for visibility, were likely to be underestimated. Foods overlaid in opaque containers can also have a considerable adverse effect on dietitians’ interpretations. We mentioned that a free-text record or double confirmation between dietitians and the participants would be needed to obtain data on actual ingredients and portion sizes. One study also supported the image-based dietary assessment, but it required minimal damage to food and low environmental interference [32]. This may be one of the main reasons that underestimation is often observed when technology-based methods are used. The wide LOA of nutrients observed in our study was similar to that of other studies. We particularly noted that carbohydrates were underestimated by approximately 15 g, and the LOA ranged from −101.52 to 71.32, as previously reported [15]. (3) Recall bias may have occurred with the written FR because participants were expected to estimate portion sizes by themselves. The parents may not realize that children’s food intake is usually less than that of adults, so they may have reported FR on the basis of their own experience, causing more overestimation in the FR method than in the photographs. We contend that photographs with annotations were highly accurate in representing the real situation because they enabled direct observation of the real portion size in food photographs.

The portability and usability of technology-based dietary assessment were major factors leading to the development of this image-based dietary assessment tool. We supposed that a dietary intake assessment tool that is compatible with smartphones is convenient for users because it can deliver messages in time, store data in a digital form, and provide regular reminders to participants to report records. During the study period, we received some feedback from the participants. Two families reported that recording images of children’s lunch was difficult because many schools disallow children from using smartphones in class. One participant said that they were reluctant to comply with the details of how to capture the photos because the process seemed to be complicated. The difficulty of component calculation was also mentioned. Although COFIT can semi-automatically determine food ingredients, many participants had difficulty reporting portion size correctly, and correct reporting of portion size is highly dependent on personal experience. To solve the aforementioned problems, we can provide a standardized china bowl, plate, and cup to use as standardized reference markers of portion size, thereby establishing the concept of portion size for the participants in future studies [21]. We encouraged children to perform the image-based dietary assessment themselves instead of asking their parents to record photographs in this study. Most of the children could accomplish the recording themselves. We believe this image-based assessment tool could reduce the participants’ burden in maintaining FR. The final goal is to assist children in long-term self-monitoring of their nutrient intake.

One of the strengths of this study was that we had an abundant nutrient database containing multiple macronutrients and micronutrients; thus, sufficient information was available for dietitians to use for analyzing nutrient intake in children of various age groups. Furthermore, it enabled dietitians to give accurate advice to the participants [16,33]. The second strength is that we used three statistical methods to validate the macronutrients and micronutrients as information assessed by COFIT and FR. However, our study was limited by non-parametric analysis under a limited sample size. A pilot study with a small sample size cannot be representative of the entire Taiwanese population. Consequently, we were not able to divide our participants into children and teenagers, since two age groups may have different food consumption habits, and especially teenagers tend to conceal what they have eaten. Additionally, we did not objectively measure biomarkers of dietary intakes, such as can be done using the DLW method, which is considered the criterion standard of measurement. Traditional dietary assessments could not record the actual effects of individual factors in the study. Another potential argument is that the differences in measurement periods may cause some systematic bias in assessing dietary intake. The FR were conducted for seven days, but the image-based dietary assessment was performed on only three days, not randomly selected by the researchers, which may be responsible for some of the differences between the FR and image-based dietary assessment.

## 5. Conclusions

Overall, this pilot study indicated COFIT is an innovative approach that makes appropriate use of technology for the collection of data on dietary intake and enables the provision of customized feedback directly to an individual. It may provide an efficient delivery method for health promotion programs that are designed for children. Recording combinations of multiple types of information and customized meal-time prompts may contribute to higher reporting frequency and accuracy; hence, both features are highly recommended for future use in technology-based dietary assessment tools.

## Figures and Tables

**Table 1:** Characteristics of the study population.

Characteristics	*n* or Mean	% or SD
Sex		
Male	13	56.5
Female	10	43.5
Age	10.47	3.36
BMI (kg/m^2^)	18.41	4.34
Thin	2	8.7
Healthy	16	69.57
Overweight	3	13.04
Obese	2	8.7

**Table 2:** Summary of macronutrients; COFIT (image-based dietary assessment) compared with written food records over three days.

	COFIT			Food Records			Wilcoxon Signed-Rank Test	^3^ SCC		
	Mean	^1^ SD	^2^ ICC	Mean	SD	ICC	*p*	*r*	95% CI	*p*
Energy (kcal/day)	1584.88	369.03	0.94	1779.11	316.29	0.85	<0.001 *	0.64	0.30–0.83	<0.01
Protein (g)	63.67	16.16	0.54	71.06	13.60	0.68	0.02 *	0.44	0.02–0.72	<0.01
Amino acid (mg)	60,229	15,835	0.83	65,140	13,262	0.73	0.10	0.50	0.11–0.76	0.01
Fat (g)	65.00	17.97	0.65	76.77	17.84	0.88	<0.001 *	0.65	0.32–0.84	<0.01
Fatty acids, total polyunsaturated (mg)	12,658.8	4961.96	0.95	13,879.95	4278.4	0.64	0.20	0.61	0.11–0.76	<0.01
Fatty acids, total monounsaturated (mg)	7418.10	2524.19	0.79	8714.20	2555.6	0.46	0.01 *	0.49	0.11–0.76	0.02
Fatty acids, saturated (mg)	6.43	2.51	0.47	7.57	3.20	0.51	0.04 *	0.54	0.17–0.78	<0.01
Carbohydrates (g)	186.50	46.15	0.98	201.59	46.82	0.67	0.08	0.47	0.07–0.74	0.03
Sugars (g)	33.48	16.78	0.87	35.27	20.85	0.89	0.60	0.67	0.35–0.85	<0.001
Glucose (g)	6.57	3.92	0.99	6.07	4.01	0.20	0.28	0.77	0.53–0.90	<0.001
Fructose (g)	7.55	4.53	0.99	5.51	3.92	0.57	<0.01 *	0.65	0.32–0.84	<0.001
Maltose (g)	1.81	1.66	0.99	1.50	0.93	0.97	0.67	0.61	0.27–0.82	<0.01
Sucrose (g)	11.28	9.04	0.57	16.13	13.48	0.97	<0.01 *	0.55	0.18–0.79	<0.01
Lactose (g)	6.30	4.79	0.95	6.28	4.58	0.98	0.93	0.94	0.86–0.98	<0.001
Dietary Fiber (g)	9.74	3.36	0.94	10.72	2.77	0.68	0.12	0.59	0.23–0.80	<0.01

^1^ SD, standard deviation; ^2^ ICC: intra-class correlation (*n* = 6); ^3^ SCC: Spearman’s correlation coefficients; * values were significantly different (*p* < 0.05).

**Table 3:** Summary of micronutrients; COFIT (image-based dietary assessment) compared with written food records over three days.

	COFIT			Food Records			Wilcoxon Signed-Rank Test	^3^ SCC		
	Mean	^1^ SD	^2^ ICC	Mean	SD	ICC	*p*	*r*	95% CI	*p*
Na (mg)	2336.28	815.92	0.92	2020.45	730.45	0.69	0.02 *	0.66	0.33–0.84	<0.001
Ca (mg)	513.21	286.49	0.83	574.76	265.53	0.79	0.34	0.48	0.08–0.74	0.02
K (mg)	1696.45	636.88	0.80	1778.35	510.31	0.87	0.60	0.45	0.05–0.73	0.03
Mg (mg)	183.33	83.11	0.82	210.90	83.34	0.63	0.27	0.28	−0.14–0.62	0.20
Zn (mg)	8.12	2.75	0.70	8.16	1.73	0.78	0.88	0.27	−0.16–0.61	0.22
P (mg)	882.39	224.64	0.68	938.26	186.53	0.94	0.30	0.43	0.02–0.71	0.04
Fe (mg)	8.92	5.75	0.88	9.97	6.43	0.73	0.70	0.52	0.14–0.77	0.01
Folic acid (µg)	188.28	56.07	0.96	202.15	56.99	0.53	0.41	0.77	0.51–0.89	<0.001

^1^ SD, standard deviation; ^2^ ICC: intra-class correlation (*n* = 6); ^3^ SCC: Spearman’s correlation coefficients; * values were significantly different (*p* < 0.05).

**Table 4:** Agreement between mean intake from energy, macronutrients and micronutrients; mean difference calculated as COFIT minus food record (*n* = 23).

	Bland–Altman Analysis (COFIT−FR)			
	Mean Difference	SD	95% LOA	
Energy (kcal/day)	−194.23	287.02	−756.79	368.33
Protein (g)	−7.39	14.72	−36.24	21.46
Fat (g)	−11.76	15.46	−42.06	18.54
Carbohydrate (g)	−15.10	44.09	−101.52	71.32
Dietary Fiber (g)	−0.97	2.91	−6.67	4.73
Na (mg)	315.84	603.43	−866.88	1499.56
Ca (mg)	−61.53	297.8	−645.22	522.16
K (mg)	−81.91	623.70	−1304.36	1140.54
Mg (mg)	−27.57	96.67	−217.04	161.90
Zn (mg)	−0.04	2.53	−5.00	4.92
P (mg)	−55.87	220.70	−488.44	376.70
Fe (mg)	−1.05	5.91	−12.63	10.53
Folic acid (µg)	−13.87	45.25	−102.56	74.82

LOA: limits of agreement. COFIT: image-based dietary assessment tool. FR: food records.

## Abbreviations

BMI	body mass index
ICC	intra-class correlation
24-HDR	24-h dietary recall
FR	food records
TEE	total energy expenditure
TPLS	Taipei Puberty Longitudinal Study
